# Fungal alkaloids mediate defense against bruchid beetles in field populations of an arborescent ipomoea

**DOI:** 10.1007/s10886-025-01578-2

**Published:** 2025-02-25

**Authors:** Alberto Prado, Susana Pineda-Solis, Roberto Garibay-Orijel, Donald Windsor, Jean-Luc Boevé

**Affiliations:** 1 Escuela Nacional de Estudios Superiores Unidad Juriquilla, Universidad Nacional Autónoma de México, Blvd. Campus Juriquilla 3001, Juriquilla, Querétaro CP 76230 México; 2 Instituto de Biología, Universidad Nacional Autónoma de México, 3er. Circuito Exterior S/N, Ciudad Universitaria, CP 04510 CDMX México; 3 Smithsonian Tropical Research Institute, Apartado 0843-03092, Balboa, Ancón, Panamá; 4 OD Taxonomy and Phylogeny, Royal Belgian Institute of Natural Sciences, Rue Vautier 29, Brussels, 1000 Belgium

**Keywords:** Defensive symbiosis, Hereditary fungi, *Ipomoea*, Swainsonine

## Abstract

Several Convolvulaceae species harbor heritable fungal endophytes from which alkaloids are translocated to reproductive tissues of the plant host. Evidence for the distribution and ecological role of these fungal alkaloids, however, is lacking or incomplete for many host species and growth forms. Here we report on the identity of the fungal endophytes and quantities of alkaloids present in the leaves and seeds of the arborescent morning glory, *Ipomoea murucoides* (Convolvulaceae). Young folded leaf samples taken from the wild, harbored mycelium of one of two fungal taxa wrapped around the leaves’ glandular trichomes. Most trees harbored the swainsonine producing *Ceramothyrium* (Chaetothyriales) fungi while a few trees were found to harbor a *Truncatella* (Xylariales) species, suggesting endophyte replacement. Seeds had higher concentrations of the indolizidine alkaloid swainsonine than leaves. Additionally, seeds from trees harboring *Ceramothyrium* fungi exhibited less bruchid damage and had higher concentrations of swainsonine than seeds from trees harboring *Truncatella* fungi. Five sesquiterpenes were detected in the leaf trichomes in both *Ceramothyrium* and *Truncatella* colonized trees. The seed content of the tropane alkaloids, tropine and tropinone, did not differ significantly among the two fungal symbionts. It is likely that the host allocates the defensive chemicals from leaves to seeds, protecting them from seed predators such as bruchid beetles. Overall, our field data show that *Ipomoea* species provides an interesting opportunity to study vertical and horizontal fungal symbiont transmissions.

**Supplementary Information:**

The online version contains supplementary material available at 10.1007/s10886-025-01578-2.

## Introduction

Plants commonly rely on defensive mutualisms for protection from herbivores. Well-studied examples include structures such as extrafloral nectaries and Beltian bodies which attract and nourish ants to defend plant foliage (Janzen 1966; Rudgers and Strauss 2004; Rico-Gray and Oliveira 2007). Other well characterized examples include grasses where microbe-mediated chemical defenses protect grass hosts against herbivory (Clay 1988). In these cases, grasses are infected systemically by endophytic fungi (Clavicipitaceae) that produce a variety of ergot alkaloid compounds deterrent or toxic to domestic mammals, and increase resistance to insect herbivores (Clay 1988; Clay and Schardl 2002). Interestingly, the next plant generation inherits these symbiotic fungi through vertical transmission in the grass seeds (Schardl 2007). The vertical transmission of fungi, heritable endosymbionts, is only known for three plant families: Poaceae, Fabaceae and Convolvulaceae. In all cases of hereditary fungi reported so far, fungal-derived alkaloids are involved (Clay and Schardl 2002; Panaccione et al. 2014; Quach et al. 2023). In the Fabaceae, *Astragalus*, *Oxytropis* and *Swainsona* species, host endophytic fungi of the genus *Alternaria* (Pleosporales) produce the trihydroxy indolizidine alkaloid swainsonine (Panaccione et al. 2014; Neyaz et al. 2022). Swainsonine protects the host tissues from grazing animals and can poison livestock (Ralphs et al. 2008). Recently swainsonine-producing fungi have been described in the pantropical family Convolvulaceae (Cook et al. 2013), where 40% of the species of *Ipomoea* are believed to host heritable fungal endophytes that synthesize chemical defenses for the plant (Quach et al. 2023). Within the genus *Ipomoea* there are lineages harboring symbiotic clavicipitaceous fungi (*Periglandula*) capable of producing ergot and indole diterpene alkaloids, while other lineages form associations with unidentified fungi belonging to the swainsonine-producing Chaetothyriales (Panaccione et al. 2014; Quach et al. 2023). Although the fungi colonize the plant systemically, epiphytic fungal colonies can be observed growing on the adaxial surface of young leaves, wrapped around the glandular trichomes that serve as oil glands (Steiner et al. 2015). This epibiotic phase is thought to be the pathway for fungal nutrition and horizontal transmission of chemical defenses. As leaves grow and unfold, fungal colonies are less visible, with mature leaves presenting no sign of colony presence or damage. Inside the seeds, the mycelium is located on the surface of the cotyledons and on the inner side of the seed coat (Pistán et al. 2022).

Swainsonine was first identified as the active principle of *Swainsona canescens*, a legume native to Australia, and later as the active principle of the “locoweeds” (*Astragalus* and *Oxytropis)* of North America (Panaccione et al. 2014). Swainsonine inhibits the enzymes alpha-mannosidase and mannosidase II, resulting in lysosomal storage disease and altered glycoprotein synthesis in the Golgi apparatus (Dorling et al. 1980). Swainsonine is a strong antifeedant for *Spodoptera littoralis* (Fellows et al. 1989) and is toxic to grazing livestock in the Americas, Asia, and Australia (Cook et al. 2009). Due to its bioactivity swainsonine is under consideration as a chemotherapy agent for certain types of cancer (Li et al. 2012; Ma et al. 2018; Singh and Kaur 2014).

Nortropane alkaloids (i.e., tropane alkaloids in which the nitrogen atom is not methylated) calystegines, and tropane alkaloids, such as tropine and tropinone, are also present in several Convolvulaceae species, although these alkaloids are believed to be produced by the plant and have been proposed as chemotaxonomic marker for the family (Schimming et al. 2005; Todd et al. 1995). Calystegines possess glycosidase inhibiting properties comparable to those of swainsonine (Schimming et al. 2005).

The identity of the swainsonine-producing fungal symbionts associated with *Ipomoea* remains unknown. Futhermore, field evidence of the potential role of swainsonine as a defensive compound is lacking. To further our understanding of the heritable endosymbionts of *Ipomoea*, in this study we aimed to (1) identify the fungal symbionts of the arborescent *Ipomoea murucoides* Roem. & Schult., (2) quantify alkaloid concentrations in leaves seeds, and (3) evaluate herbivory damage. We discuss promising questions remaining for further research.

## Methods and materials

*Sampling*. *Ipomoea murucoides* trees (*N* = 33) were georeferenced at four localities surrounding the city of Querétaro México (elevation: 1850–2400 masl). At the four localities *I. murucoides* behaves as a deciduous species, producing leaves during the summer that decay in the winter when the trees start to bloom. Fructification occurs from January to April. Young, unfolded leaves (ca. 5 g) were collected from each tree using pruning shears previously cleaned with 70% alcohol, and transported in ice to the laboratory. Dehiscent capsules were collected from the same trees (collection dates are specified in Supplementary Table 1). Seeds were removed from the capsules, cleaned from their plumose appendages and kept for 8 months under ambient conditions (28 ± 2 °C, 73 ± 3% RH) until bruchid beetle emergence. Undamaged seeds were counted and stored in envelopes for chemical analysis. Voucher specimens of plants are deposited in the herbarium QMEX (QMEX00007405 *Truncatella* harboring tree & QMEX00007406 *Ceramothyrium* harboring tree) of the Facultad de Ciencias Naturales UAQ, Querétaro, Mexico. Voucher specimens of the insects are deposited in the Insect Collection of the Facultad de Ciencias Naturales, Querétaro, México. Samples for the molecular characterization of fungi were collected during two years 2021 and 2022 on the same trees.

*Molecular characterization of fungi.* Unfolded young leaves were separated with sterile tweezers. Fungal mycelium growing on the adaxial side of young leaves was scraped with sterile spatulas and transferred to an Eppendorf tube with 600 µl of lysis buffer, following the manufacturer’s instructions (Promega DNA Wizard Purification Kit). To characterize fungi molecularly, three nuclear genes were amplified: SSU 18S using primers UF1 forward and S3 reverse (Steiner et al. 2006), ITS using ITS1 forward and ITS4 reverse (Steiner et al. 2006), and LSU 28S using LROR forward and LR5 reverse (Jeyaprakash et al. 2014). Following an initial denaturation at 94 °C for 2 min, PCR was cycled 30 times at 94 °C for 30 s, 57 °C (SSU 18S), 58 °C (ITS), 65 °C (LSU 28S) for 30 s, 72 °C for 30 s, and a final extension at 72 °C for 1 min. Polymerase chain reactions were carried out in 25 µl volumes following the manufacturer´s protocol (Promega GoTaq Flexi DNA polymerase). DNA sequencing was performed by a commercial Sanger sequencing service (Cinvestav Langebio, Irapuato, México). All sequences have been submitted to GenBank under accession numbers SSU 18S PV017332-PV017354, ITS PV023327-PV023336, LSU 28S PV050905-PV050922.

The DNA matrix for the *I. murucoides* epibiotic fungi was augmented with sequences from Genbank to include representatives of different Ascomycota fungal orders, including Hypocreales (Clavicipitaceae), Capnodiales, Chaetothyriales, Erysiphales, Eurotiales, Onygenales, Pezizales, Umbilicariales, and Xylariales. DNA sequences were edited in BioEdit v7.2 (Hall 1999), aligned with ClustalW v2 (Larkin et al. 2007) and then manually refined using Mesquite v3.02 (Maddison 2008). The concatenated DNA matrix contained 29% of missing data. The best-fit models of molecular evolution were chosen based on the Akaike information criterion (AIC) and implemented in jModelTest2 v2.1.6 using seven nucleotide substitution schemes (Posada 2008). All phylogenetic analyses were performed using the CIPRES Science Gateway v3.3 platform (Miller et al. 2010). The phylogenetic analysis involved Bayesian phylogenetic inference as implemented in MrBayes 3.2.7 (Ronquist and Huelsenbeck 2003) consisting of two independent runs of 20 million generations sampling each 1,000 generations and discarding the first 25% of the trees using a 2,220 dataset partitioned by the three genes with appropriate models applied (Supplementary Table 2).

*Glandular trichomes.* Glassware was cleaned and placed in an oven at 250 °C for 2 h. To elucidate their content, clavate glandular trichomes from ten trees were scraped off from the adaxial side of young leaves presenting no mycelium using a razor blade and avoiding rupturing the leaf epidermis. The trichomes were ground to a fine powder in liquid N_2_ using a mortar and pestle. The powder was soaked in hexane (10:1 [v/w]) containing 0.03% toluene as internal standard and extracted for 2 h at room temperature in 4 mL glass vials with tightly sealed rubber septa caps. After centrifugation at 3,000 rpm for 30 min, 0.5 mL of the clear hexane layer was transferred into to an autosampler glass vial, these extracts being stored at -30 °C. Samples were analyzed by gas chromatography-mass spectrometry (GC-MS) on Shimadzu single quadrupole GCMS-QP2020 NX using a HP5-MS 5% polysiloxane column with a tube desorption of 280 °C, flow rate of 60 mL/min, trap cooling temperature of -20 °C and a FID/MS detector splitting system. Compounds were identified by comparing their mass spectra with those from spectra libraries (NIST, FFNSC).

*Seed and leaf alkaloid concentration.* We followed the protocol of Quach et al. 2022 for swainsonine extraction. One g of seed (~8 seeds) and 5 g of fully-grown leaf tissue was extracted in 10 mL of chloroform and 20 mL of 2% acetic acid for 16 h with agitation. A 15 mL acetic fraction was transferred to a new vial and lyophilized. Lyophilates were resuspended in 5 mL of 20 mM ammonium acetate. An aliquot of 1 mL was transferred to an auto-sampler vial. Samples were analyzed by liquid chromatography-mass spectrometry (LC-MS) for swainsonine, tropine and tropinone. Standard calibration curves were prepared by serial dilutions of the commercially available standards (Sigma-Aldrich, St. Louis, MO, USA). LC-MS was carried out by a commercial service of Université Catholique de Louvain, Belgium, using a Waters Acquity UPLC BEH HILIC column (100 × 2.1 mm, 1.8 μm) with a 0.3 mL/min flow rate and a 95% ACN: 5% water with 0.1% of formic acid mobile phase. Injections consisted of 1 µl of the sample. Samples were analyzed in triplicates. The three calibration curves had r^2^ values above 0.998. The limits of detection and quantification for swainsonine were 4.3 ppb and 11.7 ppb, respectively. The limits of detection and quantification for tropine were 1 ppb and 3.3 ppb, respectively. The limits of detection and quantification for tropinone were 2.2 ppb and 7.4 ppb, respectively.

*Seed and leaf herbivory rates.* Seeds of *I. murucoides* were collected from the target trees and kept for 8 months in ambient conditions (28 ± 2 °C, 73 ± 3% RH) in the laboratory until beetle emergence (*Megacereus* aff.* tricolor* Suffrian, 1870, Bruchidae: Bruchinae). Healthy and bruchid damaged seeds were counted, while other types of damaged or malformed seeds were not. On average 134 seeds were inspected per tree. Leaf herbivory rates were estimated by clipping entire branches of *I. murucoides* foliage and placing it on the ground. We then placed a 30 × 30 cm wooden frame with a 5 × 5 cm grid above the foliage and estimated visually the percentage of tissue that had been eaten by chewing insects. Bacterial, fungal and cynipid damage were not quantified. Ten measurements were carried out per tree, and averaged.

*Data analysis.* Statistical analyses and visualization of the chemistry and herbivory data were carried out in R version 4.2.2 (R Core Team 2023). Alkaloid concentrations were compared between leaves and seeds using a generalized linear mixed model considering the tree reference code as the random factor in the *nlme* package version 3.1 (Pinheiro & Bates 2023). As the data did not follow a normal distribution, Wilcoxon rank sum tests were used to compare swainsonine concentrations in leaves and seeds in relation to fungal lineage. As the concentrations of tropine and tropinone were low and close to the limit for quantification, Fisher’s exact probability test was used on presence-absence data comparing the occurrence of these two alkaloids in leaves and seeds in relation to fungi. Wilcoxon rank sum tests were also used to compare herbivory levels of leaves and seeds in relation to the fungal symbiont.

## Results

**Fig. 1:**
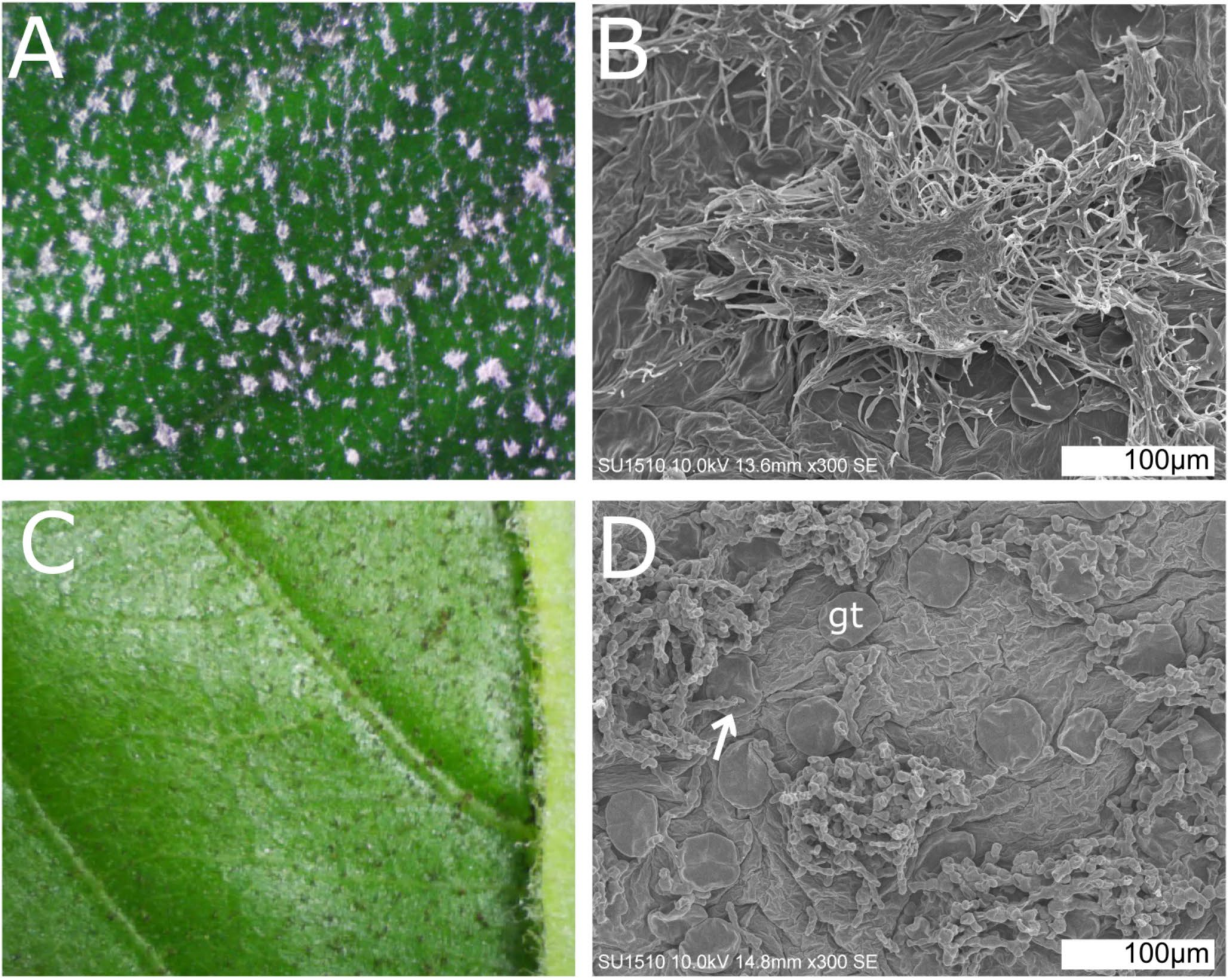
Mycelium growing around glandular trichomes of *Ipomoea murucoides*. **A**-**B** Fungi belonging to *Ceramothyrium* in the Chaetothyriaceae. **C**-**D** Fungi belonging to *Truncatella* in the Sporocadaceae. **B** & **D** Electron microscope photographs. Arrow indicates appresorium used to penetrate the glandular trichome (gt)

All the trees examined presented leaves with mycelium patches, appearing either as a white crust or as a darker covering, growing on the upper side of unfolded new leaves (Fig. 1A and C). Microscopic inspection revealed mycelium wrapped around the peltate glandular trichomes. Fungal appresoria penetrating glandular trichomes were evident in some electron microscope images (Fig. 1D). Once leaves naturally unfolded, both fungus and trichomes on the leaf surface were no longer visible, and damage to the leaf surface was never evident. The only leaves we identified not presenting mycelium were the first flushes of leaves that the tree produced in spring (the tree loses its leaves in winter). From this mycelium free-leaves we qualitatively identified five sesquiterpene compounds from the glandular trichome extracts: alpha-copaene, beta-caryophyllene, alpha-humulene, germacrene, and delta-cadinene. However, concentrations of these compounds were too low for quantification and are not shown. These five sesquiterpenes were identified in the trichomes of trees that would later present either the white or dark mycelium on their leaves.

**Fig. 2:**
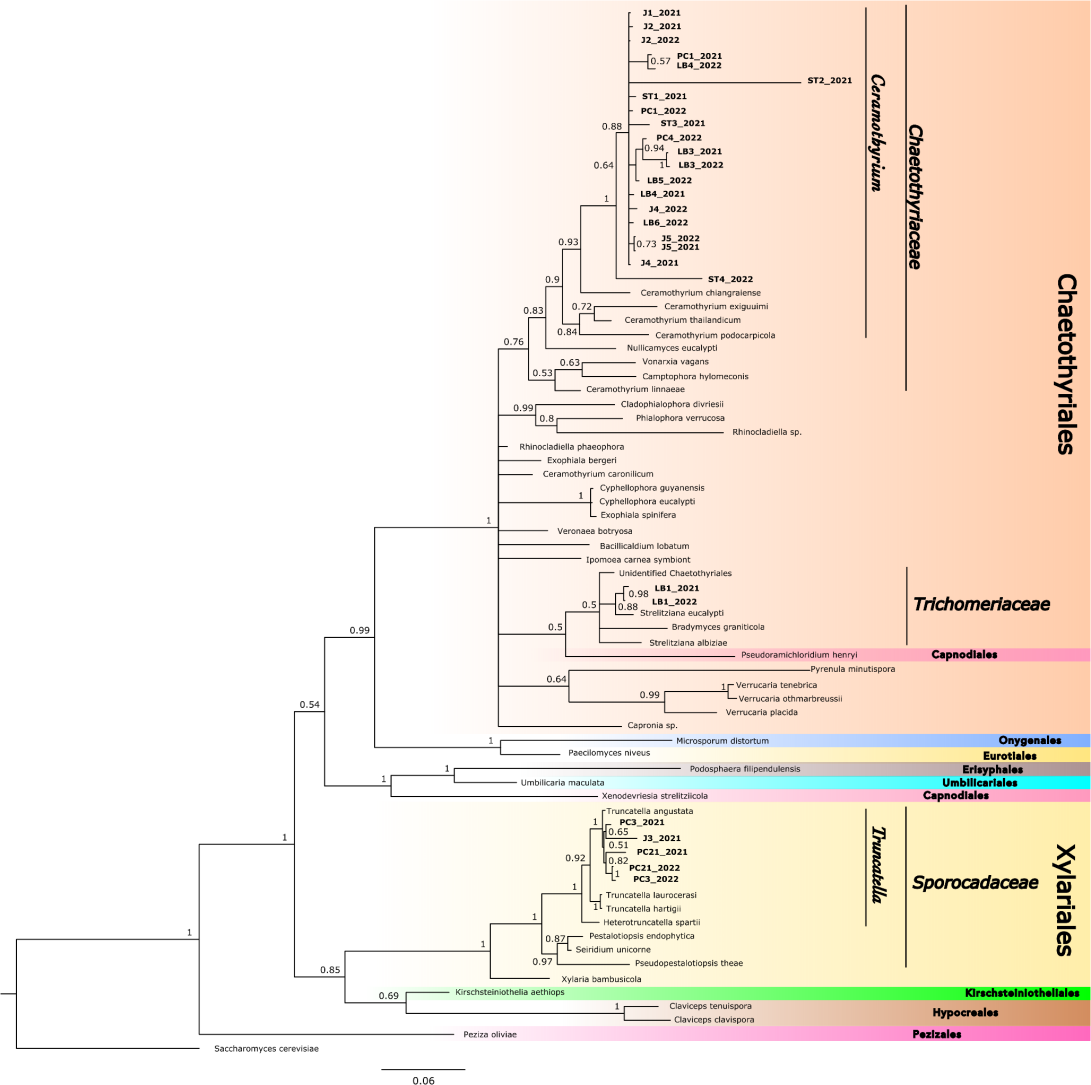
Phylogram based on a 20 million generation Bayesian analysis using 18S, ITS and 28S sequences of fungi forming symbiosis with *Ipomoea murucoides* trichomes (bold). Branch lengths are proportional to the number of nucleotide changes/site. Three lineages of fungi were identified among our field samples: *Ceramothyrium* in the Chaetothyriaceae, Trichomeriaceae (Chaetothyriales), and *Truncatella* in the Sporocadaceae (Xylariales). Sequences from *I. murucoides* symbionts are mentioned by the reference code of the tree followed by the sampling year

We identified three lineages of epibiotic fungi on *I. murucoides* leaf surfaces: two lineages belonging to the order Chaetothyriales, and a third lineage Sporocadaceae family in the Xylariales (Fig. 2). Trees with a Chaetothyriaceae fungi belonging to the genus *Ceramothyrium* were more common (*N* = 23) than trees with fungi in the genus *Truncatella* in the Sporocadaceae (*N* = 9). Only one tree presented a fungus belonging to the family Trichomeriaceae within the Chaetothyriales. The Trichomeriaceae samples were excluded from the alkaloid analyses. Over the two-year survey the identity of the fungal partner remained constant for all host trees, and none of the trees presented both families of fungi (Fig. 2).

**Fig. 3:**
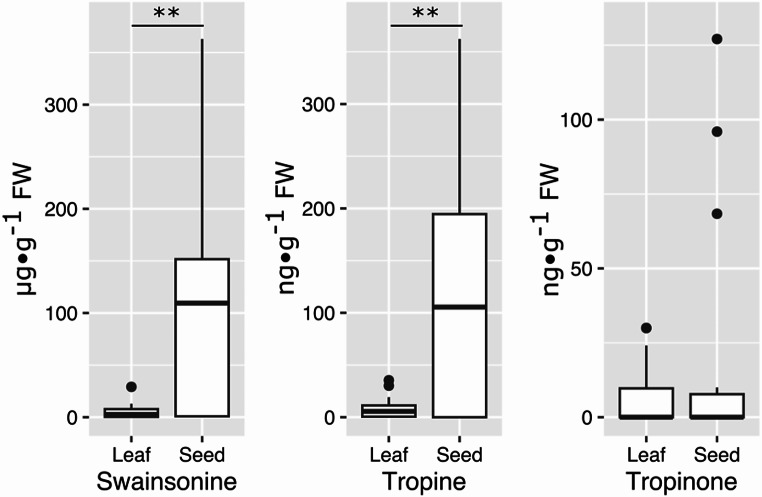
Alkaloid content of *Ipomoea murucoides* seed and leaves (mixed effect linear models considering the tree identity as a random factor, ** *p* < 0.01)

The indolizidine alkaloid swainsonine was detected in 80% of the seed samples and 83% of the leaf samples analyzed. The tropane alkaloid tropine was detected in 69% of seed samples and 72% of leaf samples. The tropane alkaloid tropinone was detected in 30% of the seed samples and 44% of leaf samples. Alkaloid concentrations were significantly higher in seeds (1–363 µg⋅g^− 1^ FW) than in leaves (0.2–29 µg⋅g^− 1^ FW; Fig. 3). Tropine and tropinone, when detected in both seeds and leaves, were at lower concentrations than swainsonine. Average tropine concentration in seeds (9–362 ng⋅g^− 1^ FW) was significantly higher than in leaves (1.9–35 ng⋅g^− 1^ FW). Average tropinone concentration in seeds (6–127 ng⋅g^− 1^ FW) was not significantly different than in leaves (2–29 ng⋅g^− 1^ FW).

**Fig. 4:**
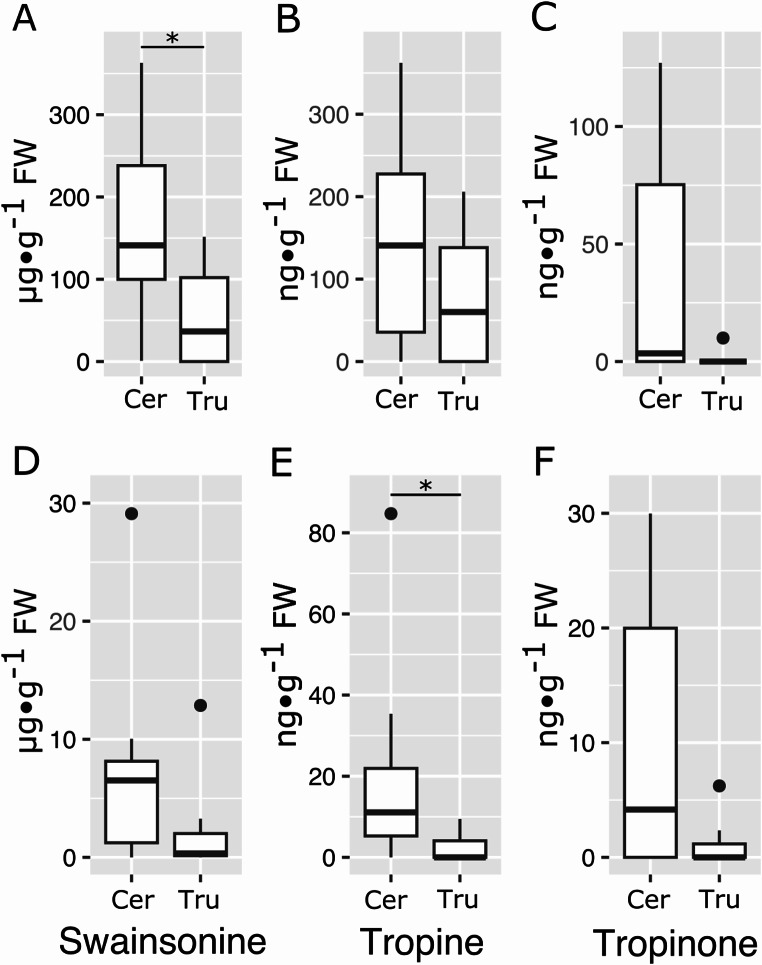
Alkaloid content of *Ipomoea murucoides* seeds (**A-C**) and leaves (**D-F**) in relation to their fungal symbiont *Ceramothyrium* (Cer), *Truncatella* (Tru). **A** & **D** Swainsonine (Wilcoxon rank sum test with continuity correction, * *p* < 0.05). **B** & **E** Tropine (Fisher’s exact probability test for count data on presence or absence, * *p* < 0.05). **C** & **F** Tropinone (Fisher’s exact probability test for count data on presence or absence)

Swainsonine concentrations were significantly higher in seed from trees with the *Ceramothyrium* symbiont (161.26 µg⋅g^− 1^) than in trees with the *Truncatella* symbiont (56.74 µg⋅g^− 1^) (Wilcoxon rank sum test with continuity correction W = 52, *p* = 0.04; Fig. 4A). The average tropine content in seeds from *Ceramothyrium* harboring trees (145.76 ng⋅g^− 1^) did not differ significantly from that found in seeds from plants colonized by *Truncatella* (77.78 ng⋅g^− 1^). Similarly, average tropinone concentrations were not significantly different between seeds from plants colonized by *Ceramothyrium* (37.30 ng⋅g^− 1^) and *Truncatella* (1.25 ng⋅g^− 1^). Swainsonine and tropinone concentrations in leaves did not differ in relation to the identity of the symbiont (Fig. 4D and E). Tropine levels were higher in leaves from trees with the *Ceramothyrium* symbiont as compared to those from trees with the *Truncatella* symbiont (Fisher’s exact probability test for count data on presence or absence, *p* = 0.047).

**Fig. 5:**
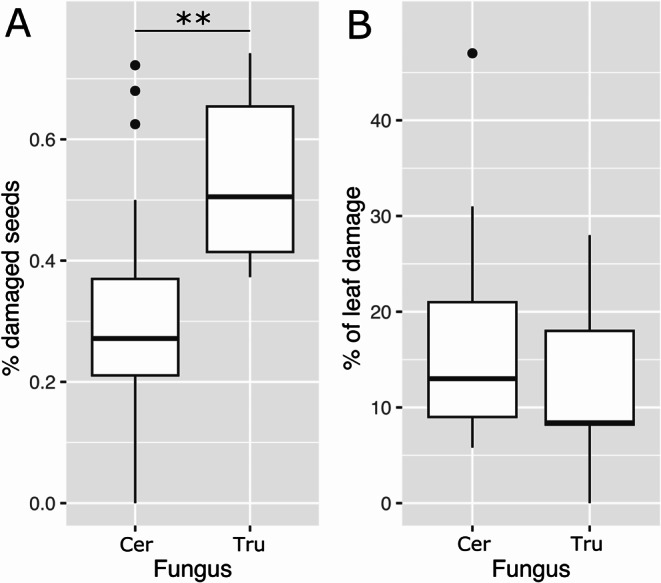
Insect herbivory rates of *Ipomoea murucoides*. **A** Granivory by *Megacerus* aff. *tricolor* (Bruchinae) and **B** leaf damage by chewing insects in relation to the trees’ symbiont *Ceramothyrium* (Cer), *Truncatella* (Tru). Wilcoxon rank sum test with continuity correction, ** *p* < 0.01

Seeds of *I. murucoides* were heavily damaged by the beetle *M. aff. tricolor*. The beetle’s activity accounted for up to 75% of seed loss in the localities we studied. Seeds from trees harbouring *Ceramothyrium* exhibited less bruchid damage than trees harboring *Truncatella* (Fig. 5A). Herbivory of the leaves by chewing insects - mainly two Chrysomelidae species: *Ogdoecosta mexicana* Champion, 1893 and *Charidotella bifossulata* Boheman, 1855 - did not differ between trees with different fungi symbionts (Fig. 4B).

## Discussion

Plants have been traditionally considered as the “ultimate chemists” with more than 200,000 plant secondary metabolites described (Hartmann 2007). However, endophytic microorganisms have been recognized as playing an important role in plant chemistry (Strobel el al. 1996; Steiner et al. 2006). Plant hosts may benefit from these microbial synthesizers as the acquired compounds confer ecological advantages. Since several microbial compounds have been shown to deter insects (Clay et al. 1985; Cook et al. 2014; Grum et al. 2013), insect herbivory/granivory could be a key driver in the evolution of endophyte-mediated chemistry (Clay 2014).

This study determined that the arborescent *I. murucoides* is colonized by fungi belonging to two orders, Chaetothyriales and Xylariales, the former being prevalent. Based on the DNA sequences of the 18S, ITS and 28S regions, we have putatively identified the genera involved as *Ceramothyrium* in the Chaetothyriaceae and *Truncatella* in the Sporocadaceae. Fungi developed on the inside surface of young, folded leaves where they interacted with glandular trichomes. The folded nature of the leaf possibly serves as protection from the dry environment. Five sesquiterpene compounds were identified in extracts of the glandular trichomes of *I. murucoides.* These five compounds were reported from the glandular trichomes of *I. asarifolia*, which harbors a clavicipitaceous fungal symbiont of the genus *Periglandula* (Steiner et al. 2015). The lipophilic oil products of the trichomes are thought to serve as a food source for the fungal symbionts (Steiner et al. 2015). For some *Ipomoea* species, a mucilaginous secretion consisting of polysaccharides is produced by the glandular trichomes of young-folded leaves (Kuster et al. 2016). The mucilaginous secretion on young leaves promotes the adherence of the two leaf blade lobes, and therefore reduces the leaf area in direct contact with the external environment (Kuster et al. 2016). However, such a secretion could potentially also serve as food supply for the fungi. In this study we did not search for the presence of such a mucilaginous secretion. Besides their secretory capacity, the glandular trichomes are thought to provide a site for entry of fungal alkaloids (Steiner et al. 2015). We quantified swainsonine in the leaves and seeds of trees harboring both symbionts. A higher concentration of swainsonine was observed in the seeds as compared with leaves, which may indicate a translocation of the chemical defenses between tree organs. Such a transfer from leaves to seeds has been suggested for the ergot alkaloids acquired from *Periglandula* fungal partners through the leaf trichomes of *I. asarifolia* and *I.* (*Turbina*) *corymbosa* (Steiner et al. 2015). Due to its deciduous behavior,* I. murucoides* is devoid of leaves when the tree is in bloom. Hence the translocation of alkaloids from leaves to seeds would require an intermediate storage step in the stems or roots. Translocation of swainsonine* in Astragalus lentiginosus* has been suggested to occur via the phloem (Dreyer et al. 1985). As* I. murucoides* leaves unfold, mycelium and trichomes fall off the leaf blade leaving no signs of damage. In this study we quantified alkaloids in fully grown leaves devoid of epibiotic mycelium. We did not evaluate how leaf age affects alkaloid concentrations.

Interesting parallels exist between the *Periglandula/Ipomoea* and the *Ceramothyrium/Ipomoea* symbioses. In both systems alkaloids are involved and the glandular trichomes seem to play a key role. Why *Ipomoea* is predisposed to form such symbioses with fungi remains unclear. Among 244 studied species of *Ipomoea*, the 32 species which tested positive for swainsonine in their seeds belong to a single clade within the *Ipomoea* phylogeny (i.e., clade A1 in Wood et al. 2020; Quach et al. 2022). The arborescent group of *Ipomoea* of central Mexico all reside in the A1 clade and are all suspected to harbor fungal symbionts. Ergot alkaloids and indolizidine diterpene alkaloids are present in other clades of the *Ipomoea* phylogeny in a discrete distribution pattern (Quach et al. 2023). The phylogenetic signal in the distribution of alkaloids in the *Ipomoea* genus suggests that these symbiotic relationships are relatively conserved in time, and likely the result of vertical transmission of the symbionts. However, 30% of *I. murucoides* individuals we studied were associated with a different type of fungi belonging to the genus *Truncatella*. Seeds from these individuals also contained swainsonine but at lower concentrations. The swainsonine gene cluster has been identified in both the Chaetothyriales and Xylariales fungi (Neyaz et al. 2022), which could explain the presence of these alkaloids in all analyzed individuals. However, swainsonine production has never been confirmed in the Xylariales, so an alternative explanation to the presence of swainsonine in *Truncatella* host trees, is that the symbiont replacement is incomplete, and a fraction of the tree still harbors the true swainsonine-producing *Ceramothyrium* symbiont. The symbiont switch (or partial switch) in certain trees is best explained by a horizontal transmission where the *Truncatella* fungi replaced *Ceramothyrium*, but it remains an open question requiring future research. We consider that *I. murucoides* provides an excellent research system for exploring vertical and horizontal fungal transmissions.

The fact that tropinone was absent in so many samples could be a result of its role as an intermediary in calystegine biosynthesis (Scholl et al. 2001). However, we did not quantify calystegines in our study. Calystegines are thought to contribute to the toxicity of *Ipomoea* species (Cook et al. 2013), hence their quantification should be included in future chemical surveys of *I. murucoides*. Furthermore,* I. murucoides* is known to contain other unidentified metabolites with insecticidal properties; leaf hexane extracts have been shown to cause* Spodoptera frugiperda* mortality (Ocampo-Antonio et al. 2022).

Our results support the hypothesis that the toxicity of alkaloids derived from fungal mutualists lowers morning glory losses to specialist, seed-feeding bruchid beetles. This mutualism is probably not involved in the protection of leaves as the concentrations of compounds are extremely low and has negligible effects on rates of herbivory by specialist chrysomelid beetles. Further, trees associated with a different fungal symbiont from the family Sporocadaceae produced lower concentrations of swainsonine and suffered greater seed losses from bruchid beetles. This evidence supports the ecological value of defenses in the Convolvulaceae mediated by fungal symbiosis.

## Electronic Supplementary Material

Below is the link to the electronic supplementary material.

Supplementary Material 1

Supplementary Material 2

## Data Availability

Data is provided within the manuscript or supplementary information files.
